# A Consulting Institute

**Published:** 1899-05-20

**Authors:** 


### A CONSULTING INSTITUTE.

There is one aspect of the proposed Consulting Institute in Birmingham to which attention may well be drawn, for it would seem that it is the intention of the founders to establish a system which is comparable only with that of the medical aid associations which now occupy such a discredited position in the eyes of the profession. According to the account given to the annual meeting of the Birmingham Hospital Saturday Fund it would seem that the plan is that rooms should be taken in a central position, and two consultants, a physician and a surgeon, should be engaged, for one year certain, at a salary of £500 per annum each. It was stated that the fee suggested was half a guinea for each consultation, not to include medicine, and that the hours would be fixed to meet the habits of working men. The expenditure for the first year was estimated at about

£1,500, and it was thought that 20 consultations a day might be obtained, which would yield an annual income of £3,000, "and leave, after payment of all expenses, a considerable profit." Now, this manner of dealing with medical men—farming out their services and pocketing the difference between the fees earned by the doctors, "consultants" in this case, and their salaries —is exactly what is done by the medical aid associations, and is exactly one of the causes of offence which leads the profession at large to look askance at the medical officers of such institutions, and it will be a matter of some interest to observe how the profession will regard those young men who may be seduced by the bait of £500 a year and the name of "consultant" to accept office at this institution. In a letter to the Birmingham *Daily Gazette*, Dr. Saundby has very properly pointed out that a "highly qualified and clever young man" is by no means a "consultant" as the word is understood at the present time, however much he may consult. He says: "There are in every quarter of this town medical practitioners who were, when they started, highly qualified and clever young men, but they elected to earn their living as general practitioners. Any one of these is well qualified to give all that the consulting institute could afford, and I have no doubt under existing circumstances would give it for five shillings, and certainly for half a guinea, to anyone who would consult them." This is true. Nothing could be easier than to set up a consulting institute. But will the patients care to pay their half guineas to see these young men? for "highly qualified and clever young men," who often find it difficult enough to get even half-a-crown for good honest work, are by no means scarce. The founders of this notable scheme seem to have overlooked the fact that the very essence of consultation practice is repute, and that the public care nothing for the opinion of a man who is unknown.

